# A novel mutation I522N within the *TGFBI* gene caused lattice corneal dystrophy I

**Published:** 2009-11-28

**Authors:** Chunmei Zhang, Guang Zeng, Hui Lin, Dandan Li, Liming Zhao, Nan Zhou, Yanhua Qi

**Affiliations:** Department of Ophthalmology, Harbin Medical University the 2nd Affiliated Hospital, Harbin, China

## Abstract

**Purpose:**

To identify mutations within the *TGFBI* gene in a Chinese family with lattice corneal dystrophy type I (LCD I).

**Methods:**

Genomic DNA of three affected, four unaffected family members and 50 normal individuals was extracted from peripheral leukocytes. All exons of *TGFBI* were amplified by polymerase chain reaction (PCR) methods and direct sequencing was carried out for mutation analysis.

**Results:**

A missense mutation (1565T→A) in exon12 of *TGFBI* led to an amino acid substitution I522N in the TGFB-induced protein in all affected family members, but the mutation was not detected in normal subjects of the family and control individuals.

**Conclusions:**

We conclude that the novel mutation I522N causes lattice corneal dystrophy type I in the studied family. This is the first report of the I522N mutation within *TGFBI* in LCD I worldwide.

## Introduction

Corneal dystrophies are hereditary diseases characterized by corneal opacities on different layers of the cornea. Lattice corneal dystrophy is known as an autosomal dominant disease. Histological examination of corneal specimens shows amyloid deposits. The typical clinical appearance of LCD I (LCD I; OMIM 122200) is characterized by subepithelial and stromal branching lattice lines. Generally, the clinical symptoms become evident in the patient’s first or second decade, with the appearance of white-grayish opacities in the superficial stromal layer of the cornea. Thereafter, the lesions tend to become larger, aggregate, and extend deeper and toward the periphery over time.

The transforming growth factor-beta-induced gene (*TGFBI*) is located on the human chromosome 5q31 [1]. Munier et al. [1] reported that mutations in *TGFBI* caused lattice corneal dystrophy type Ι. To date, the mutations reported in *TGFBI* as the causation of LCD type Ι include R124C, V505D, L518P, V539D, A546D, P551Q, L569R, H572R, and V625D [2-4]. The following mutations in *TGFBI* causing LCDI have been identified in Chinese families**:** R124C, V625D, and V505D [5,6]. In this study, we described a novel mutation I522N in *TGFBI* inducing LCDI in a Chinese family. None of the previously reported mutations in the *TGFBI* gene were found in this family.

## Methods

### Patients

**Figure 1:**
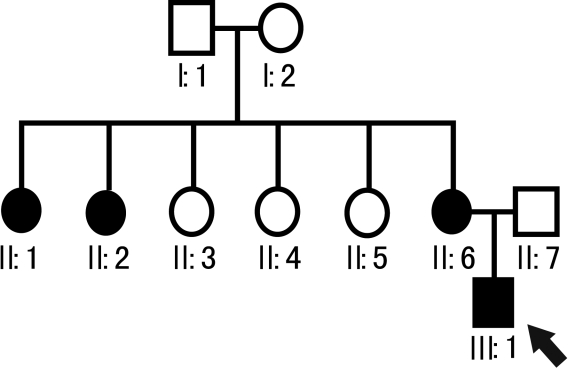
Pedigree analysis and pedigree symbols. The squares indicate males and the circles indicate females. A filled symbol indicates a person affected with LCD I. The arrow indicates the proband.

This study was approved by the Institutional Review Board of Harbin Medical University (Harbin, China). Four affected and six unaffected individuals from a Chinese family (Figure1) were enrolled in this study after obtaining informed consent. Fifty unrelated healthy individuals were selected as the control group and they were all Chinese. All subjects, including control individuals, underwent clinical ophthalmologic examination and slit lamp photographs of affected eyes were taken.

### Molecular genetic analysis

Peripheral blood (5 ml) was taken from patients, unaffected family members, and 50 healthy controls. Genomic DNA was extracted from peripheral leukocytes, according to the manufacturer's (Tiangen Biltech Co. Ltd, Beijing, China) standard methods. All 17 exons of *TGFBI* were amplified by polymerase chain reaction (PCR) using the primers described previously [7]. PCRs were performed in a 50 μl volume containing 10× PCR buffer, 10–200 ng of genomic DNA, 0.2 mM of each dNTP, 1 unit of Taq polymerase, and 1 μl of 1 mM forward and reverse primers. The primer annealing temperature was adjusted separately for each PCR reaction, which was based on those described by Li et al. [7]. After pre-denaturation at 95 °C for 5 min, DNA fragments were amplified for 35 cycles of denaturation, annealing, and extension, followed by a final extension step at 72 °C for 10 min. PCR products were analyzed in 2% agarose gel, from which the bands with the amplified templates were examined and subsequently purified with a TIANgel Midi Purification Kit (Tiangen Biltech Co. Ltd) and sequenced with an ABI BigDye Terminator Cycle Sequencing kit v3.1 (ABI Applied Biosystems, Foster City, CA). Nucleotide sequences of PCR products were manually compared with NCBI *TGFBI* Gene Reference Sequences (NM_000358.2).

## Results

### Clinical findings

**Figure 2:**
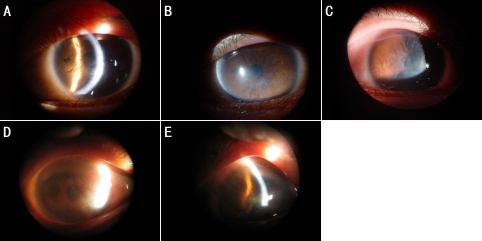
Clinical photographs of affected family members with LCD I. **A** and **B**: Slit lamp appearance of a cornea of a proband at 16 years of age shows distinct refractile lattice lines and diffuse opacification in the subepithelial and anterior stromal layer in the left eye. **C**: The photograph demonstrates central anterior stromal clouding in the proband’s right eye. **D**: The photograph shows lattice opacities with the appearance of new vessels in the subepithelial and anterior stromal cornea of the proband’s mother, with recurrent disease in the right eye. **E**: The image of the proband’s mother reveals thick linear opacities and diffuse grayish-white clouding, which partly covers the original lattice lines within the central area of the cornea in the left eye.

The common feature of corneal lesion in the pedigree was that the onset of the disease occurred in adulthood, approximately in the second decade of life, and is characterized by ocular pain, photophobia, and progressive visual defect. Clinical data of patients from the family were shown in Table1. Slit lamp examination revealed typical symmetric branching lattice lines in the central anterior stroma of the proband. The proband's mother showed new corneal vessels with recurrent corneal erosion and multiple thick subepithelial, stromal lattice opacification (Figure2). All of the affected individuals were referred to appropriate specialists for a work-up for systemic amyloidosis. However, no features of this discovery were detected.

**Table 1:** Clinical data for the affected members in the family with the *TGFBI* I522N mutation.

**Individual case**	**Age**	**Age at onset**	**Visual acuity at presentation**	
			**OD**	**OS**
III-1	16	12	0.3	0.4
II-6	38	13	0.06	0.12
II-1	46	15	HM/50 cm	HM/30 cm
II-2	43	13	0.1	HM/50 cm

The Snellen visual acuity scale was used in these studies. In the table, HM indicates hand movement.

### Gene analysis

**Figure 3:**
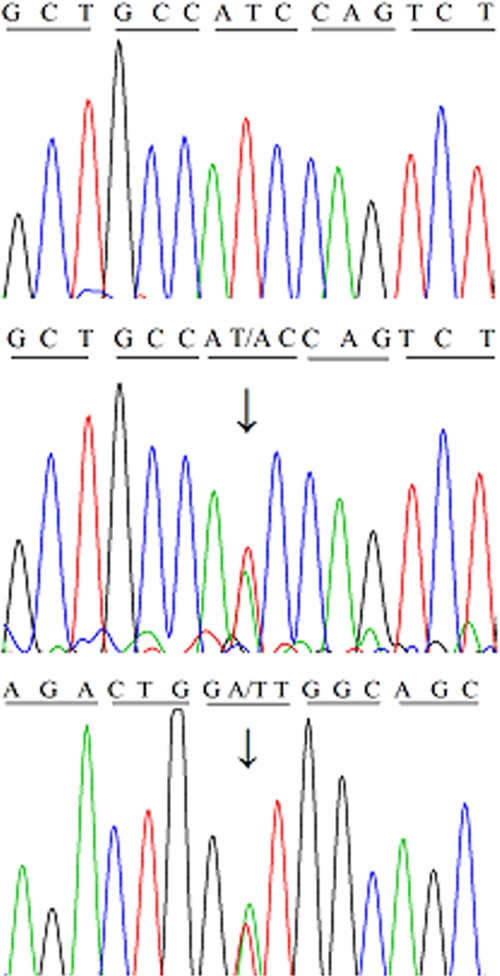
Partial nucleotide sequence of exon 12 of *TGFBI* in affected and unaffected individuals. **A**: A normal sequence is shown where the isoleucine is encoded by codon 522. **B** and **C**: The forward and reverse sequence shows a heterozygous genotype (1565T→A) in all the affected family members, which resulted from a change of isoleucine (ATC) to asparagine (AAC) at codon 522, thus altering the structure and property of the protein.

**Figure 4:**
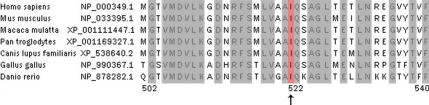
Protein sequence of TGFBI in diverse species. The alignment of the TGFBI sequence with the corresponding segments among several species is shown. The isoleucine is conserved in the TGFBI protein in diverse species. The reference sequences were obtained from the *TGFBI* Gene.

Exon 12 of *TGFBI* of the affected and unaffected individuals was analyzed by direct sequencing (Figure3). The sequence of the patients revealed a heterozygous T→A mutation at nucleotide 1565. This nucleotide substitution resulted in an amino acid replacement from isoleucine (ATC) to asparagine (AAC) at codon 522 (I522N). The reverse sequence of the mutation showed a corresponding base replacement A>T, which further confirmed the mutation. No other non-pathogenic SNPs were observed in the other remaining *TGFBI* exons of the family. The homologous sequences within the TGFBI protein from diverse species were listed in Figure4. The alignment shows that the mutation I522N is located at a highly conserved site.

## Discussion

In 1992, *TGFBI* (*BIGH3*) was first isolated by Skonier et al. [8] in a study of genes induced by the transforming growth factor-ß (TGFß) in a human adenocarcinoma cell line derived from the lung. *TGFBI* (*BIGH3*) was found to be expressed in the corneal epithelium [9]. So far, all the pathogenic mutations reported on *TGFBI* are only related to corneal dystrophies, which are characterized by progressive accumulation of deposits in the cornea [10]. It has been shown that corneal deposits are made of the mutant KE protein [11]. The genetic studies revealed specific missense mutations in *TGFBI*, causing diverse hereditary corneal dystrophies. The most frequent types of these dystrophies result from mutations at two hot spots, which are localized at codons Arg124 and Arg555 [12].

The *TGFBI* gene product is a secreted protein of 683 amino acids, consisting of four regions of internal homology of approximately 140 amino acids and a carboxy terminal Arg-Gly-Asp (RGD) sequence. The tandem repeated regions are known as FAS1 domains. These four FAS1 domains correspond to amino acids 134-236 (FAS1-1), 242-372 (FAS1-2), 373-501 (FAS1-3), and 502-632 (FAS1-4) [13]. All the mutations of *TGFBI* that cause corneal dystrophy are located in FAS1 domain 4, with the exception of Arg124 [13]. Clout et al. [14] made a homology model of the FAS1 domain 4 of *TGFBI* protein, which suggested the common mutations of *TGFBI* at positions 124 and 555 are likely to affect protein-protein interactions directly, whereas the rare mutations are likely to cause misfolding of the protein within the cell.

The mutation described in this study is located in residue 522 and in the fourth FAS1 as well. This residue is conserved among several species and thought of as a distinct functional and/or structural sequence of the protein induced by TGFB gene. According to the model of Clout et al. [14], Ile522 is a hydrophobic residue and situated in helix α1. The mutation alters highly conserved residue and leads to the substitution of hydrophilic residue for hydrophobic residue. The nucleotide replacement may alter the protein's physico-chemical property and affect proper folding of the protein within the cell, which results in abnormal corneal deposits and corneal dystrophy.

To date, five types of LCD have been established according to the stromal location of deposits, the characteristics of lattice lines, and the age of onset. The five types are I, II, III, IIIA, and IV [15]. Only LCD type II is caused by mutations in the GSN gene, while the others are all linked to mutant *TGFBI* and different phenotypes resulting from distinct mutation points in the same gene. In addition, several atypical forms of LCD, which can not be classified as LCD I, II, III, IIIA, and IV, have been observed to be associated with various *TGFBI* mutations [16-18]. Genetic testing of *TGFBI* mutations in LCD patients will contribute to the clinical classification, correct diagnosis, prevention and treatment level of hereditary disease. For those individuals, genetic testing at an early age may improve clinical outcomes by encouraging frequent ocular examination and thus laying a solid foundation for genetic therapy.

This is the fourth genetic mutation reported in association with the classic type I of LCD. The novel gene mutation expands the mutation spectrum of *TGFBI* and contributes to the study of molecular pathogenesis of corneal dystrophy.
